# Centralised versus local measurement of glycated haemoglobin in clinical trial settings: a comment on Arch et al., *Trials*. 2016

**DOI:** 10.1186/s13063-017-1944-1

**Published:** 2017-05-15

**Authors:** Sudip Chatterjee, Richeek Pradhan

**Affiliations:** 1 Department of Medicine, Vivekananda Institute of Medical Sciences, 99, Sarat Bose Road, Kolkata, West Bengal 700026 India; 2 Park Clinic, Kolkata, India

## Abstract

Arch and colleagues in their 24 October 2016 paper in *Trials* focus on the issue of centralised versus local measurement of glycated haemoglobin (HbA1c) in clinical trial settings. Resolution of the debate is important: while local HbA1c measurement is less costly, and would thereby ease the stretched funding situations for clinical trials worldwide, it cannot be implemented at the expense of clinically unacceptable disparities between centralised and localised measurements. Arch and colleagues favour centralised measurement in their paper’s conclusion. However, critical questions regarding the methods require a closer look. In this letter, we discuss some of the issues that the authors could clarify in order that the reader can agree (or disagree) to their inference with greater confidence.

## Background

Arch and colleagues [1] report findings in an area of interest in diabetes clinical trials regarding glycated haemoglobin (HbA1c) measurement which we have explored earlier [2]. The paper concludes, ‘Variation in agreement between HbA1c measurements was greater than had been expected…’, and recommends that ‘centralised HbA1c measurement is preferable in the multicentre clinical trial setting’. Some of the evidence adduced bears a closer inspection.

1. Finding of lack of correlation between time-lag and difference of local and central measurements
  The authors conclude a lack of correlation between time-lag and difference of local and central measurements by reporting a Spearman rho of *r* = −0.02 (confidence limits not mentioned). If we look at Figure 3, we see that the median (local-central) HbA1c measurements from five centers are above zero, and from four centers are below zero, while six are too close to call. Taken together, the mean HbA1c difference reported is 0.16 mmol/mol, 95% CI −0.20, 0.52 (Table 3). In such equal dispersion around zero, is it not expected that an *r* with time-lag will also be close to zero? Can we, given this information, infer lack of correlation? Our experience was that, depending on the method used, HbA1c can vary significantly with a time-lag between sample collection and measurement. This was particularly so when the sample had unusually high glucose values. A scatter plot showing the relationship between time-lag and HbA1c measurement discrepancy may help the reader infer with greater confidence.
2. Inadequate details regarding HbA1c measurement methodology
  Discrepancies in measurements of HbA1c may depend on the method used centrally and locally, as well as on the conditions that the samples are subjected to during transfer. While the authors mention that the biochemical methodology mentioned for ‘almost all’ HbA1c measurements (local and central) were immunoassays, they do not specify the local methods used in the 15 trial sites. Immunoassay techniques vary in their efficiencies of removing labile HbA1d from the sample, a common artifact contributing to falsely elevated HbA1c values. Again, from Fig. 3, the reader can infer that even the local methods differ from each other. Moreover, ambient temperature may have a role in discrepancies resulting from time-lag between sample collection and measurement [3]. The paper mentions that the transportation was done by two methods (by post, or by bespoke courier systems), but does not elaborate on quality control during transportation. Without these details, the reader could question the cause of the discrepancy between local and central laboratory measurements.

## Conclusions

Our findings were that the time between sample collection and testing in a central laboratory could alter the HbA1c value if the ambient glucose levels were high. This was partly due to the inefficiencies of the proprietary methods in removing HbA1d from the samples. In the paper by Arch et al., 93% percent of the differences between local and central measurements were within clinically acceptable limits. This, combined with our findings of a discrepancy between central and local measurements, strengthens the case for local standardised methods to measure HbA1c in diabetes trials.
